# Norwegian Endurance Athlete ECG Database

**DOI:** 10.1109/OJEMB.2022.3214719

**Published:** 2022-10-21

**Authors:** Bjørn-Jostein Singstad

**Affiliations:** Department of Computational PhysiologySimula Research Laboratory 394155 Kristian Augusts Gate 23,0164OsloNorway

**Keywords:** Electrocardiograms, athletes, dataset, physionet

## Abstract

Athletes often have training-induced remodeling of the heart, and this can sometimes be seen as abnormal but non-pathological changes in the electrocardiogram. However, these changes can be confused with severe cardiovascular diseases that, in some cases, can cause cardiovascular death. Electrocardiogram data from athletes is therefore important to learn more about the difference between normal athletic remodeling and pathological remodeling of the heart. This work provides a dataset of electrocardiograms from 28 Norwegian elite endurance athletes. The electrocardiograms are standard 12-lead resting ECGs, recorded for 10 seconds while the athlete's lay supine on a bench. The electrocardiograms were then interpreted by an interpretation algorithm and by a trained cardiologist. The electrocardiogram waveform data and the interpretations were stored in Python Waveform Database format and made publicly available through PhysioNet.

## I. Introduction

Athletes often have increased thickness in the left ventricular wall and extended chambers in both the left and right ventricles compared to untrained people at the same age. These structural changes can be difficult to distinguish between normal athletic remodeling of the heart, also known as athletes heart [1], and severe cardiovascular diseases (CVD), such as hypertrophic cardiomyopathy (HCM), dilated cardiomyopathy (DCM), arrhythmogenic right ventricular cardiomyopathy (ARVC) and left ventricular noncompaction (LVNC). These pathologies are associated with a risk of Sudden Cardiac Death (SCD). Due to the low prevalence of these diseases, it requires very high precision in order to minimize the number of false positives.

Currently, there exist specific criteria for interpreting electrocardiograms (ECG) from athletes [2]. The interpretation criteria aim to detect athletes at risk of having a sudden cardiac arrest, but also point out fewer false positives. The latter is important because many false positives will represent a heavy burden on the health care system and an unnecessarily high mental burden on the incorrectly interpreted individual athlete [3].

The interpretation criteria for athletes are constantly evolving, and Berge et al. 2015 [4] showed how the Seattle criteria from 2013 [5] lowered the number of abnormal ECG findings from 29.3% (specified European Society of Cardiology recommendations [6]) to 11%. Furthermore, Refined Criteria (2014) [7] has been shown to lower the number of false positives further, and at the same time not lower the detection rate of sick athletes [2], [8]. On the other hand, it requires a lot of expertise to interpret these ECGs correctly, and in addition, these criteria are based on manual interpretation, which can be very time-consuming for the interpreter [9].

An alternative to manual interpretation is computer-based interpretation. A study compared the interpretation of ECGs from athletes using algorithms versus visual measurements performed by specialists and identified limitations of algorithm-based ECG interpretations on athletes [10]. On the other hand, new methods such as AI-based ECG interpretation have shown promising performance in the last couple of years [11], [12], [13], [14], [15] and these methods might improve today's ECG interpretation algorithms. In order to train AI-based algorithms, data is needed, and this paper presents the first open-access ECG database containing ECGs from elite endurance athletes. Therefore, this article marks the beginning of an open-source development of artificial intelligence based ECG interpretation algorithms for athlete cohorts.

## II. Data Collection Procedures

### A. Participants

The participants who donated their ECG to this study were informed and gave written consent before the data acquisition was initiated, they also agreed to have their ECG shared in an open database. The study protocol and consent form were approved by the Norwegian Centre for Research Data (application ID: 389013) and the University of Oslo. The ethical considerations were approved by the Regional Committees for Medical and Health Research Ethics (application ID: 51205).

**Figure 1.**
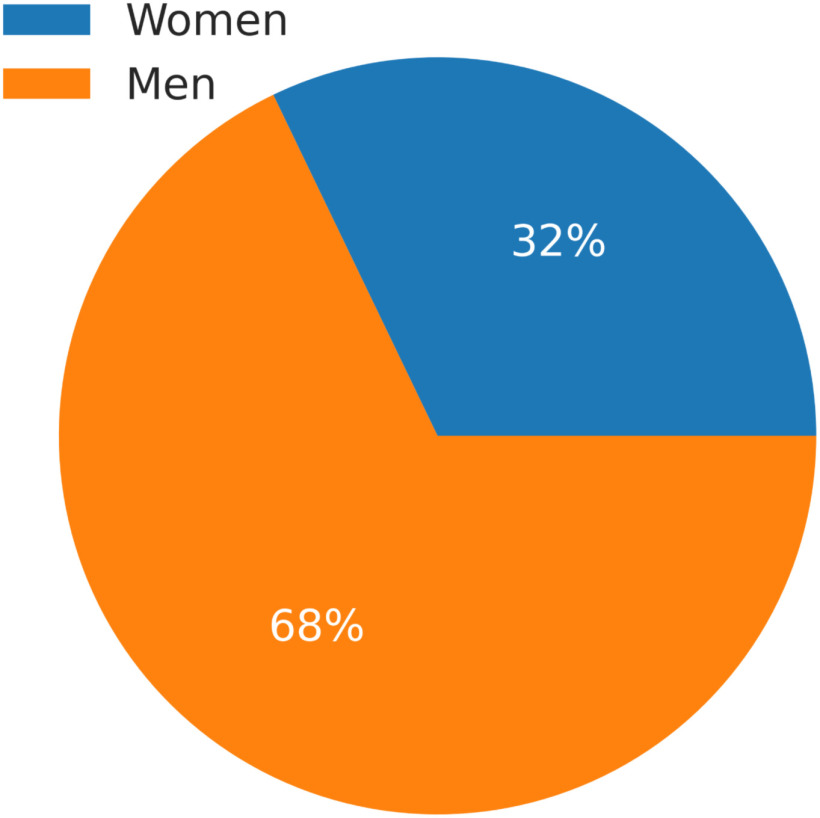
The diagram shows the distribution between men and women in this dataset.

Twenty-eight healthy athletes were recruited for this study. From Fig. 1 we see that 19 (68%) of the participants were men and 9 (32%) were women. Participants' ages ranged from 20 to 43 years (Mean = 25 years, standard deviation = 4.7 years). The distribution among sports was 24 rowers (86%), 2 kayakers (7%) and 2 cyclists (7%). The average amount of training hours for 2017 was 822 hours with a standard deviation of ±117 hours, in 2018 the average amount of training was 820 hours with a standard deviation of ±113 hours and in 2019 the average amount of training was 798 hours with a standard deviation of ±171 hours.

Athletes from the sports cycling, rowing and kayaking were invited to this study to increase the chance of including athletes with athletic remodeling of the heart. Studies show that athletes performing mainly static or isometric exercise, like weightlifting have primarily developed increased left ventricular wall thickness with unchanged left ventricular chamber size [16]. Purely dynamic endurance sports, like running, typically results in increased left ventricular chamber size with a proportional increase in wall thickness [16]. While cycling, rowing and kayaking involve a combination of endurance- and strength [17], a meta-analysis of athlete's hearts showed that cyclists and rowers had a significant increase in relative wall thickness and the highest increase in left ventricular internal diameter compared to strength and dynamic endurance athletes [16].

### B. Signal Acquisition

**Figure 2.**
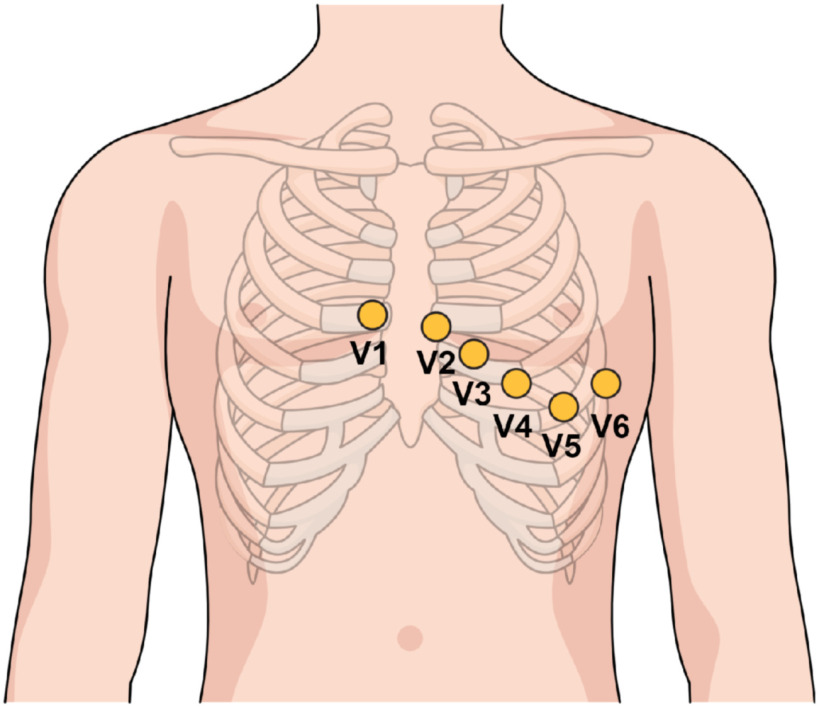
The figure shows how the precordial leads were placed on the test subjects. The illustration is made using Mind the Graph (https://mind-thegraph.com/).

**Figure 3.**
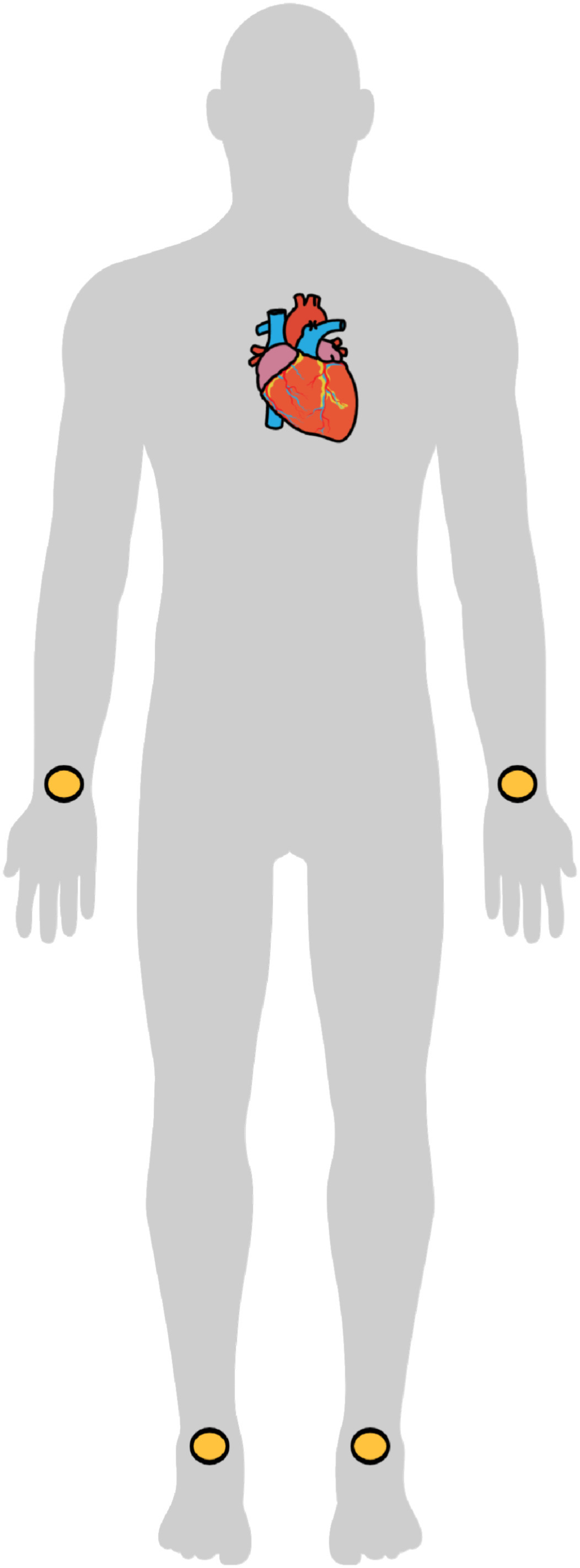
The figure shows how the limb leads where placed on the the subjects in this study. The illustration is made using Mind the Graph (https://mindthegraph.com/).

The test subjects were lying horizontally on a bench, relaxing, while electrodes were attached to perform a 12-lead ECG recording. The precordial leads were attached to the chest as shown in Fig. 2, and the limb leads were placed on the wrists and ankles as shown in Fig. 3. The electrodes used were of the type Ambu© BlueSensorQ from Ballerup in Denmark.

The recordings were performed as a standard 10 seconds resting ECG and sampled with a sampling frequency of 500hz. The device used to record the ECGs was a GE MAC VUE 360.

### C. Interpretation

**Figure 4.**
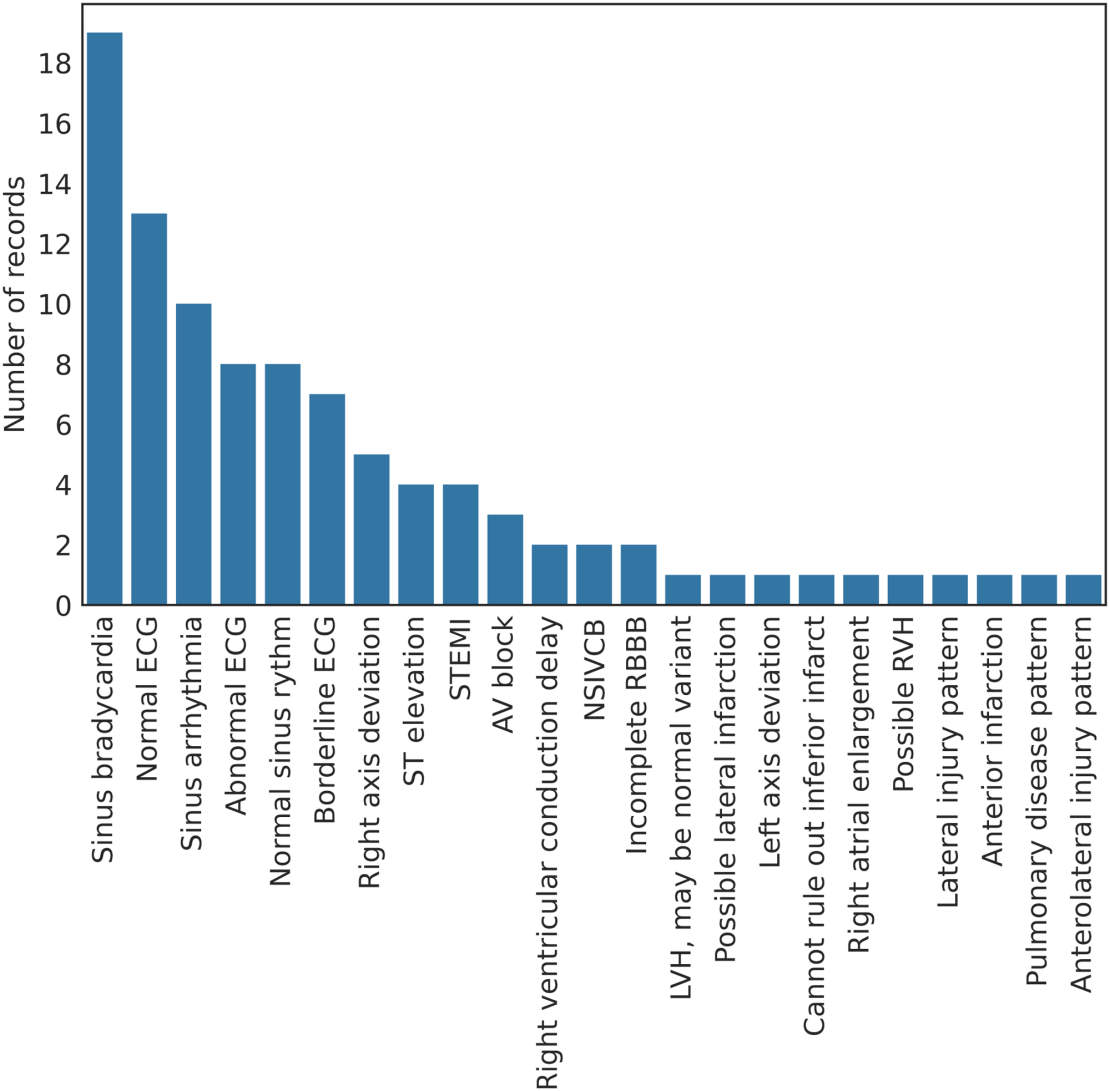
The bar plot represents the prevalence of each diagnose (on the y-axis), given by the GE Marquette SL12 algorithm.

**Figure 5.**
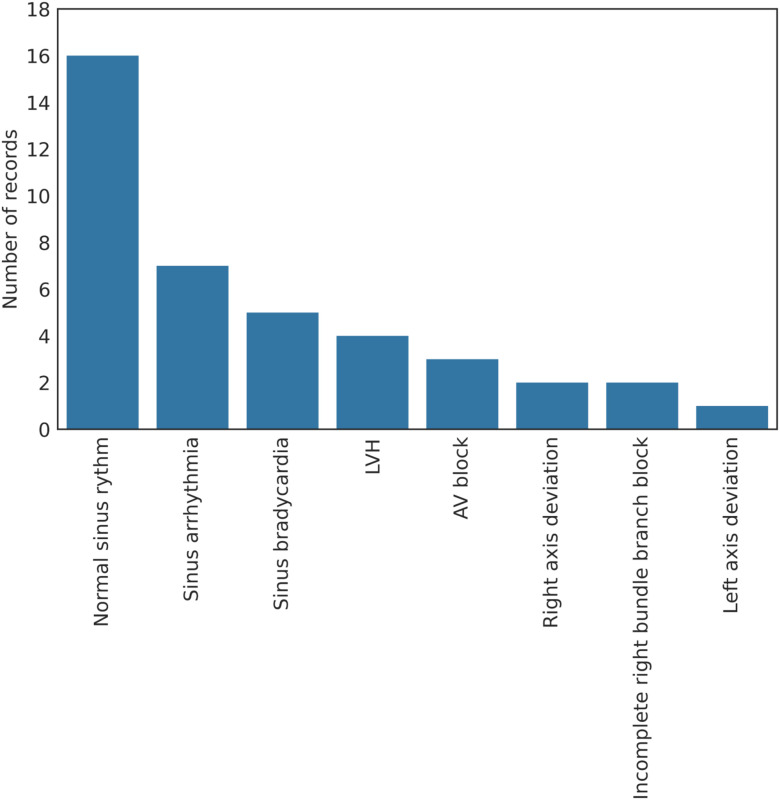
The bar plot represents the prevalence of each diagnose (on the y-axis), given by the cardiologist.

GE MAC VUE 360's built-in interpretation algorithm, Marquette 12SL (version 23 (v243)), performed an automatic interpretation of all ECGs after each recording was taken. The interpretations given by the Marquette 12SL algorithm are summarized in Fig. 4. Each ECG was automatically stored in the memory of the GE MAC VU360 device as a .ecg file format. The files were transferred via USB to a standalone PC where CardioSoft (Version V6.73) was used to convert the .ecg files to XML files. Furthermore, all ECG recordings were examined by a cardiologist, with a specialization in athletes' hearts. This cardiologist was given access to all the ECG recordings in PDF format. The PDF document includes both signals from the 12 leads and interpretive texts from GE Marquette SL 12. The cardiologist interpreted the ECGs according to the international criteria for ECG interpretation of athletes [2] The interpretations given by the cardiologist are summarized in Fig. 5.

## III. Dataset Description

The ECG recordings and interpretations from the cardiologist and the built-in software in the electrocardiograph are stored and published in a repository in the Physionet Database.^1^^1^The Physionet repository for Norwegian Endurance Athlete ECG Database: https://physionet.org/content/norwegian-athlete-ecg/1.0.0/ In addition to the dataset and a description of the dataset, this repository contains some example code in Python to make it easy to get started with this dataset.

### A. Data Records

The waveform data is given in a binary format without any modifications or additional filtering other than what's performed in the GE hardware. The ECG files were retrieved from the GE apparatus using a USB stick. The files were saved in an encrypted GE proprietary file format and in order to extract the raw ECG data GE CardioSoft was used to convert the .ecg files to XML files. The raw ECG waveform and the interpretation text from the built-in algorithm were then extracted from the XML files and stored in .dat and .hea files. The raw ECG recordings were stored in .dat -files with a corresponding .hea file containing all the metadata for the corresponding recording. These file formats are compatible with the WaveForm DataBase (WFDB) package and this makes it convenient to import the data to Python [18]. Each of the 28 .dat files consists of a 12 x 5000 array, where 12 is the number of leads and 5000 is the number of samples in each lead. The header file contains information about the total amount of leads, samples per lead and additional information about each lead. The last two lines in the header file contain the diagnosis given by the Marquette SL12 (SL12) algorithm and the cardiologist (C). An example of such a header file is shown in Table 1.

**TABLE 1:** Header File Containing Meta Data About the Current Measurment and the Test Subject

ath_001 12 500 5000
ath_001.dat 16 50000/mV 16 0 10251 49595 0 I
ath_001.dat 16 50000/mV 16 0 -1096 35223 0 II
ath_001.dat 16 50000/mV 16 0 -10267 60826 0 III
ath_001.dat 16 50000/mV 16 0 -3724 3505 0 AVR
ath_001.dat 16 50000/mV 16 0 9391 26379 0 AVL
ath_001.dat 16 50000/mV 16 0 -5395 57481 0 AVF
ath_001.dat 16 50000/mV 16 0 13580 61759 0 V1
ath_001.dat 16 50000/mV 16 0 11410 33501 0 V2
ath_001.dat 16 50000/mV 16 0 14721 52508 0 V3
ath_001.dat 16 50000/mV 16 0 16103 51083 0 V4
ath_001.dat 16 50000/mV 16 0 6662 44197 0 V5
ath_001.dat 16 50000/mV 16 0 -3806 11333 0 V6
#SL12: sinus bradycardia with marked sinus arrhythmia, Right Axis Deviation, Borderline ECG
#C: Sinus arrhythmia, Normal ECG

An example of a metadata file. The first line of the table shows the recording number (ath_001), number of ECG leads (12), sampling rate in hertz (500), number of samples (5000). The next 12 rows shows the filename containing the corresponding waveform data (ath_001.dat), the number of bits used to write the data (16), the amplitude resolution in in mV, the resolution of the analog-to-digital converter (16), baseline value (0), the first value of the signal, the checksum and the 12 lead names. In the last 3 rows, the prediction by the GE Marquette SL12 algorithm (#SL12) and the cardiologist (#C) are given.

### B. Technical Validation

The cardiologist who interpreted the ECGs also investigated the ECG waveforms to assess if there were misplaced electrodes. The cardiologist found misplaced electrodes in one case, and this is pointed out in the interpretation text in the .hea file.

## IV. Usage Notes

The intended use of this database is for the development of data-driven algorithms designed to make predictions based on ECGs. One interesting case would be to use this cohort as a test set for a trained machine learning model, in order to see how the model performs on this group of healthy athletes with relatively low heart rates and possibly also cardiac remodeling. A lot of work on machine learning and ECGs has been done on open ECG datasets in conjunction with machine learning challenges [19], [20]. These datasets [21], [22], [23], [24], [25], [26] are mainly collected in a clinical setting and therefore a huge part of the patients would have certain diseases. This dataset, however, represents a healthy population with some special properties which might also are seen in unhealthy patients and therefore cause the machine learning model to fail.

Another use case would be to blend the ECGs in this database with ECGs from other databases and use it as a training set for a data-driven model. This would potentially give a more diverse data set and increase the robustness of the model.

Due to the relatively low numbers of ECGs and subjects in this dataset compared to many other ECG datasets [21], [22], [23], [25], [26], it could be beneficial to do some data augmentation or synthetic data generation based on the present dataset. One possibility would be to train a generative adversarial network [27], [28] on the ECGs in this database, and potentially be able to generate new, synthetic ECGs with athletic properties.

One of the unique features of this database is that the ECGs are annotated by both a trained cardiologist and by state-of-the-art ECG software. Thus, it is possible to compare new interpretations with state-of-the-art ECG software and a skilled cardiologist.

### A. Limitation

The ECGs were recorded from elite athletes performing rowing, kayaking and cycling. These three sports are categorized with the highest requirements for both strength and endurance capacity, which increase the chance of developing an athletic heart. However, there might be differences in the likelihood of developing athletes’ hearts between those three sports as well. It is therefore important to bear in mind that this dataset is skewed, with 86% of the subjects being rowers, and this might have an impact on the properties of the ECGs. Aggregated statistics on this dataset might represent elite rowers more than elite athletes in general.

No echocardiographic or other examinations were performed to investigate the structure of the heart. Therefore, despite the measurements being taken from top-trained athletes, it is not confirmed whether they had athletic remodeling of the heart or not.

The cardiologist who investigated the ECGs concluded that nothing pathological was detected in the athlete's ECG. These athletes are therefore considered to be a healthy cohort. However, there will not be any follow-up studies on these athletes, and therefore we will not know for sure if any of the athletes developed diseases at a later stage.

## V. Code Availability

The data set is available on the following webpage: https://physionet.org/content/norwegian-athlete-ecg/1.0.0/.
